# P300 amplitudes in the concealed information test are less affected by depth of processing than electrodermal responses

**DOI:** 10.3389/fnhum.2012.00308

**Published:** 2012-11-15

**Authors:** Matthias Gamer, Stefan Berti

**Affiliations:** ^1^Department of Systems Neuroscience, University Medical Center Hamburg-EppendorfHamburg, Germany; ^2^Department of Psychology, Johannes Gutenberg-University MainzMainz, Germany

**Keywords:** concealed information test, memory, depth of processing, N200, P300, skin conductance, mock crime

## Abstract

The Concealed Information Test (CIT) has been used in the laboratory as well as in field applications to detect concealed crime related memories. The presentation of crime relevant details to guilty suspects has been shown to elicit enhanced N200 and P300 amplitudes of the event-related brain potentials (ERPs) as well as greater skin conductance responses (SCRs) as compared to neutral test items. These electrophysiological and electrodermal responses were found to incrementally contribute to the validity of the test, thereby suggesting that these response systems are sensitive to different psychological processes. In the current study, we tested whether depth of processing differentially affects N200, P300, and SCR amplitudes in the CIT. Twenty participants carried out a mock crime and became familiar with central and peripheral crime details. A CIT that was conducted 1 week later revealed that SCR amplitudes were larger for central details although central and peripheral items were remembered equally well in a subsequent explicit memory test. By contrast, P300 amplitudes elicited by crime related details were larger but did not differ significantly between question types. N200 amplitudes did not allow for detecting concealed knowledge in this study. These results indicate that depth of processing might be one factor that differentially affects central and autonomic nervous system responses to concealed information. Such differentiation might be highly relevant for field applications of the CIT.

## Introduction

The valid differentiation of offenders and people who are innocent of a crime under investigation is one of the most important issues in forensic sciences. Correspondingly, there has been a huge interest in developing adequate techniques that allow for such detection (Vrij, 2008). One such line of research has focused on questioning techniques and different interrogation methods have been proposed that all have distinct advantages and drawbacks. A method that seems to be widely accepted in the scientific community is the so-called Guilty Knowledge or Concealed Information Test (GKT, CIT; Lykken, 1959; Ben-Shakhar et al., 2002). This method can be regarded as an indirect test for an involvement in a crime since the participant is not asked accusatory questions (e.g., “Did you rob the grocery store last night?”) but instead confronted with a series of questions presented in a multiple choice format that ask for specific details of the crime under investigation (e.g., the weapon that was used for the robbery, the amount of money that was stolen). Each question consists of the known critical detail (relevant or probe item) and a number of neutral alternatives (irrelevant items) that are equally plausible to an innocent person. It is assumed that the recognition of crime related details by a guilty examinee results in enhanced physiological responses as compared to the irrelevant items. Innocent examinees without such knowledge should show a non-systematic response pattern. Indeed, this general response pattern has been found for a number of different behavioral and physiological measures (for a comprehensive overview see Verschuere et al., 2011).

Traditionally, CIT examinations were designed to measure autonomic responses, and the majority of studies relied on skin conductance as the only dependent measure (Ben-Shakhar and Elaad, 2003). Guilty examinees normally respond to the presentation of the crime related detail by showing enhanced skin conductance responses (SCRs). However, respiratory suppression and heart rate deceleration were also found to reliably differ between probes and irrelevant CIT items when the examinee is able to recognize crime related information (for a review see Gamer, 2011). With respect to measures of central nervous system activity, the majority of CIT studies focused on event-related brain potentials (ERPs). In most of these studies, a third item type (so-called targets) was introduced to the examination protocol (e.g., Farwell and Donchin, 1991; Allen et al., 1992; Rosenfeld et al., 2004; Meijer et al., 2007; Mertens and Allen, 2008). These target items require a different behavioral response than all other items and were used to maintain the subject's attention during the testing procedure. ERP-studies on the CIT consistently reported larger P300 amplitudes following the presentation of probes as compared to irrelevant items (for a review see Rosenfeld, 2011). Moreover, a few recent studies also reported larger N200 amplitudes when examinees were confronted with previously encoded information (Matsuda et al., 2009; Gamer and Berti, 2010). Taken together, autonomic responses as well as ERP measures allow for validly identifying concealed knowledge.

The majority of studies either focused on autonomic responses or ERPs but did not directly compare response patterns of these two types of measures. Only in recent years, a few studies tried to overcome this limitation and acquired autonomic and ERP measures within the same CIT setting (Matsuda et al., 2009, 2011; Ambach et al., 2010; Gamer and Berti, 2010). These studies mainly replicated what had already been established for separate measurements but also provided some evidence for an incremental validity of these measures. Thus, a combination of P300 amplitudes and autonomic responses enhanced CIT validity above each single measure (Ambach et al., 2010; Matsuda et al., 2011). A comparable result was obtained for the combination of N200 amplitudes and electrodermal measures (Gamer and Berti, 2010). What these studies did not address, however, is the underlying cause of this incremental validity.

At least three explanations seem plausible: First, the reliability of each single measure might be corrupted by an unknown amount of error. Assuming that this error is not substantially correlated between measures, a combination would always yield higher validity coefficients because of an increase in the signal-to-noise ratio. Second, physiological measures might be differentially responsive to concealed information or the pattern of responsiveness might vary between individuals (Matsuda et al., 2006). Such physiological effects might explain the previously observed enhanced validity for a combination of measures. Third, it seems possible that different physiological measures cover partially different psychological processes that are involved in the CIT (e.g., attentional orienting, memory retrieval, response selection, and monitoring). This would also explain the incremental validity of individual responses as well as the usually observed low to moderate correlations between measures (Matsuda et al., 2009; Gamer and Berti, 2010). Of course, these explanations are not mutually exclusive and incremental validity could rely on all these aspects. In the present study, however, we focus on the last explanation of incremental validity.

The current study was designed to shed further light on a psychological factor—namely depth of processing—that might have a differential influence on electrodermal and electrophysiological responses: It is a well established finding that items are better remembered when they have been processed more elaborately. In a seminal study on this issue, participants had to classify words according to specific criteria (e.g., typescript, rhyme, or meaning) that required a different depth of processing (Craik and Tulving, 1975). In a surprise memory test, participants were much better in remembering the initially presented words when they had been processed deeply during the encoding phase. From these data, it seems likely that memory for certain (mock) crime details also depends on depth of processing during crime execution. Since the CIT basically resembles a memory test, comparable differences should also be evident in the physiological data that is acquired during the CIT examination.

Two recent studies examined this hypothesis and tested whether autonomic responses during the CIT differ between central items that were encoded deeply during the mock crime as compared to more peripheral details which were only shallowly encoded (Nahari and Ben-Shakhar, 2011; Peth et al., 2012). Items were defined as central when they were directly related to the execution of the crime or actively handled during the course of the mock crime (e.g., the stolen item). By contrast, peripheral details were also present at the crime scene but unrelated to its execution (e.g., a picture hanging on the wall). Consistent with the hypotheses on the influence of depth of processing, participants remembered central details much better in these studies and also showed stronger autonomic responses to central items as compared to more peripheral details (c.f., Carmel et al., 2003). However, it is difficult to infer whether the latter effect is only an epiphenomenon of the reduced recognition rate or represents a specific effect of depth of processing on the autonomic level. Evidence for the latter interpretation comes from a recent study by Gamer et al. (2012). In this study, a dissociation was observed between autonomic responses and an explicit memory test: In detail, the results showed larger SCRs to deeply encoded details while no differences were obtained in an explicit memory test. Comparable results were also reported by Ambach et al. (2011) who found enhanced autonomic responses to stolen items as compared to details that were only seen during the mock crime. This result emerged even when excluding items that were not explicitly remembered but it only occurred when emphasizing the act of stealing in the CIT questions. Taken together, it seems that electrodermal responses might be sensitive to depth of processing. A study by Ferlazzo et al. (1993) suggests that this also applies to the P300 amplitude: In this study, P300 amplitudes in the test phase were enhanced for items which were deeply processed in the study phase compared to shallowly processed items. In the context of the CIT the effect of depth of processing on the P300 amplitude was not directly tested. However, previous studies consistently reported differences in P300 amplitudes between recognized items and irrelevant details for different domains including autobiographical or other personally relevant knowledge (e.g., Rosenfeld et al., 1995), mock crime details (e.g., Farwell and Donchin, 1991; Abootalebi et al., 2006), or explicitly learnt items (e.g., van Hooff et al., 1996). Moreover, it has been reported that P300 amplitudes were larger for highly salient autobiographical information (such as one's own name) as compared to incidentally acquired knowledge (such as the experimenter's name) (Rosenfeld et al., 2006). Similar results were obtained when comparing autobiographical information to explicitly learnt items (Ellwanger et al., 1996) or incidentally acquired mock crime details (Rosenfeld et al., 2007). Assuming some similarity between autobiographical information and deeply processed knowledge, it can thus be speculated that depth of processing also affects P300 amplitudes in a CIT protocol. However, the autobiographical information that was used in previous CIT studies (e.g., the own name, birthday, home town or school) was highly salient and could therefore be identified as personally relevant without difficulties. Such knowledge might qualitatively differ from other information that is only personally significant because it is linked to a specific episode in life. To further examine how the strength of episodic memories relates to physiological responses in the CIT, the current study focused on information that was encoded in the same situation (a mock crime) but with varying depth of processing (central vs. peripheral details).

Taken together, the current study was designed to test whether depth of processing differentially affects electrodermal and ERP responses in a CIT. A mock crime procedure was constructed that involved the incidental encoding of two central and two peripheral items which were all relatively salient in order to heighten the probability of successful item recognition during the CIT. To overcome limitations of previous studies using a compromise between the requirements for a reliable quantification of ERPs (i.e., large number of stimulus repetitions) and SCRs (i.e., long interstimulus-intervals, ISIs), we implemented a new approach that has been originally developed for event-related functional magnetic resonance imaging (fMRI) studies. In this kind of studies it becomes highly problematic or even impossible to detect experimentally induced changes in slow fluctuations of the blood oxygen level dependent signal when using very short fixed ISIs. Dale (1999) demonstrated that this problem can be solved by properly jittering the ISIs and optimizing the sequence of events to ensure adequate randomization. Such an approach substantially enhances the statistical efficiency of rapid event-related fMRI designs. Because the problem experienced by such fMRI studies is very similar to the problem of reliably quantifying stimulus-related SCRs with short ISIs, we transferred the approach developed by Dale (1999) to the current study in order to simultaneously measure SCRs and ERPs within the same experiment.

## Materials and methods

### Participants

Twenty-two subjects volunteered to participate in the study and gave written informed consent according to the Declaration of Helsinki. Two participants were excluded. In one case, the electrodermal data was lost due to technical problems. For another subject, the EEG-data could not be analyzed due to excessive blinking. The final sample consisted of 20 persons (19 right-handed) of whom 13 were female and 7 male. Most participants were students and their mean age was 24.5 years (SD = 6.3 years). All subjects were paid 10,-EUR for their participation.

### Procedure

All participants were instructed to accomplish a realistic mock crime scenario where all critical details (printed in *italics* below) were only incidentally encoded during the mock crime itself and not mentioned in the instructions. Participants were given a room number and were asked to find this specific room in the psychology department. Upon entering the room (an *office*), they were instructed to search for a key to unlock a desk drawer. A *keyring pendant* was affixed to this key that was always placed on the desk and could thus be easily found. Participants were instructed to “steal” a data storage medium (a *CD*) from the desk drawer. They were told to have a look at the content of the CD at home and send a short message to an email address that was given to them in advance. In this email, participants should briefly describe what they found on the CD and attach all files. There were six *pictures* on the CD that depicted a woman entering a store and leaving it with an envelope in her hands. The photos were taken from a perspective that looked like an observation of the respective woman. All participants correctly accomplished the mock crime and gave a valid description of the CD content in their email.

Following previous studies (Gamer et al., 2010; Nahari and Ben-Shakhar, 2011), we defined half of the relevant crime details as central and peripheral, respectively. Items that must have been perceived in order to successfully accomplish the mock crime were designated as central details (the CD and the content of the CD). The other details (the office where the mock crime took place and the keyring pendant) were not directly relevant for the mock crime itself and might or might not have been encoded during the course of the mock theft. Therefore, they were defined as peripheral. Thus, the question type (central vs. peripheral) was varied as a within-subjects factor.

Participants were instructed to return to the laboratory one week after execution of the mock crime to take a polygraph test. Before the examination, they were informed that a theft occurred in the psychology department a week ago. Because they were seen in the building at that time, they were told to be suspects of this theft but they would have an opportunity to demonstrate their innocence in a polygraph test examination. To increase motivation, all participants were encouraged to convince the polygraph examiner of their innocence and it was announced that all participants who successfully passed the CIT would have a chance to win 100,- EUR in a subsequent lottery.

**Figure 1:**
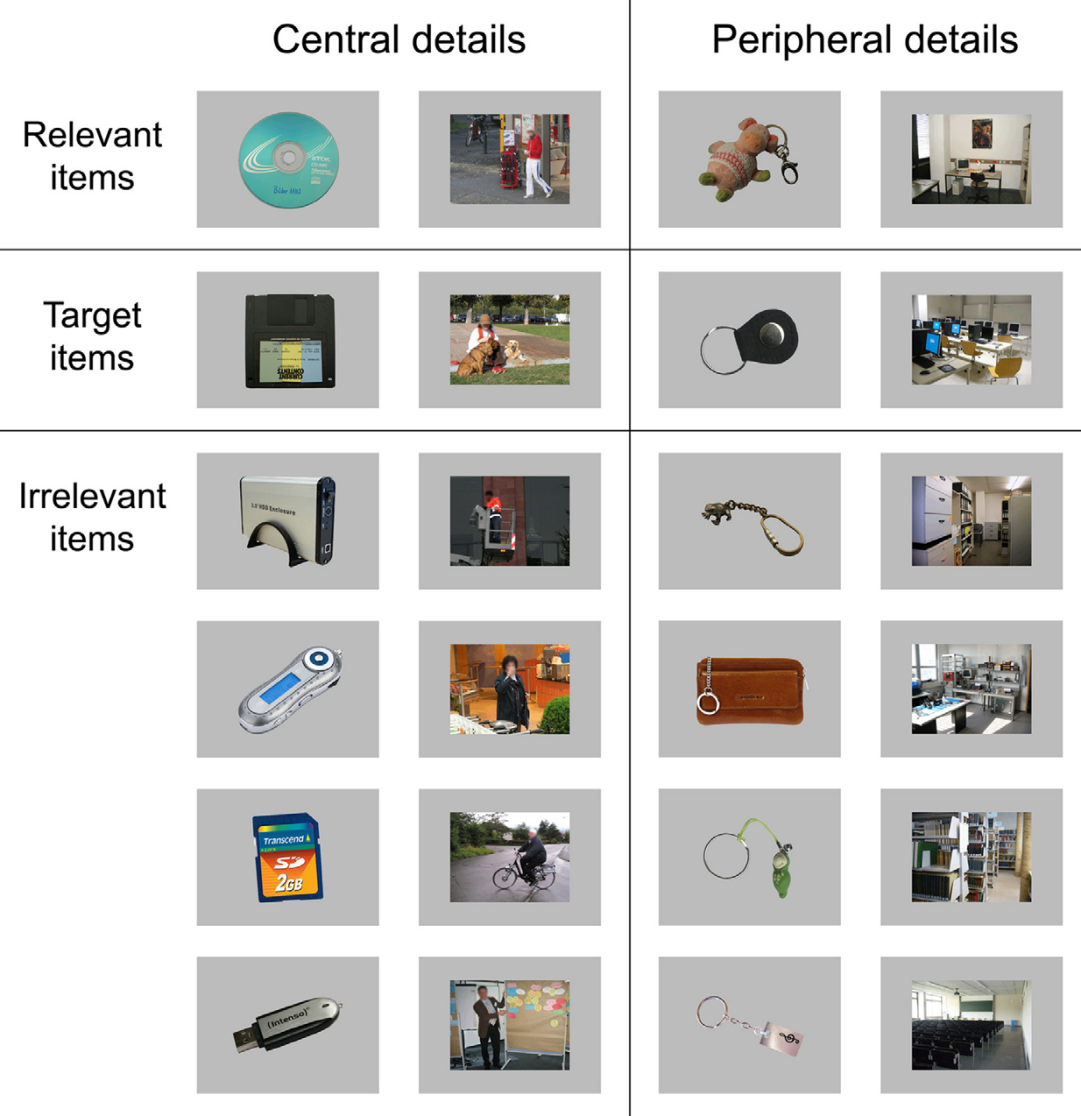
**List of all stimuli that were presented during the Concealed Information Test (CIT) in the current study.** Note that all faces were blurred for this illustration to prevent identification of the depicted persons.

For the polygraph examination, the participant was seated in a semi-reclining chair approximately 130 cm in front of a 19′ color screen in an electrically shielded and sound attenuated chamber. The stimuli were presented as pictures with a size of 16.0 × 12.1 degrees of visual angle for duration of 1000 ms each (all stimuli are shown in Figure 1). An IBM-PC using the ERTS stimulation software (BeriSoft, Germany) controlled stimulus presentation. The examination was conducted according to the 3-item CIT protocol that includes probes (the crime related details), irrelevant items (equally plausible neutral details) and targets (Farwell and Donchin, 1991; Rosenfeld et al., 2004; Meijer et al., 2007). Target items are similar to irrelevant details with the exception that they require a different behavioral response to ensure that participants are paying attention to the stimulus presentation. Therefore, participants had to memorize four specific target items (i.e., one for each CIT question defined by the respective probe item) before the examination started. Participants were instructed to press the right key of a response pad with the middle finger of their right hand whenever a target item would be presented on a display screen. The left key had to be pressed with the right index finger following all other stimuli. That is, probe and irrelevant items shared the same response key. It was emphasized that key presses should be as accurate and fast as possible.

The two central and two peripheral probe items were presented in separate blocks whose order was counterbalanced across participants. Within each block, four irrelevant items and one target were additionally presented for each probe item and each item was shown in 15 trials. The blocks were divided into three sessions each with short breaks in between to prevent tiredness. Each session started with the presentation of an additional irrelevant item that was used as a buffer and discarded in the analyses. Furthermore, the amount of trials of each item category (irrelevant, probe, target) was constant across sessions. Altogether, 30 probe (two relevant details × 15 repetitions), 30 target (one target for each probe item × 15 repetitions) and 120 irrelevant items (four irrelevant items for each probe item × 15 repetitions) were presented for central and peripheral CIT questions, respectively.

Stimulus sequence and timing were optimized using the software optseq2 (http://surfer.nmr.mgh.harvard.edu/optseq/). This software was originally developed to optimize the statistical efficiency of event-related fMRI studies (Dale, 1999). However, since the hemodynamic response that is measured by fMRI has a similar response morphology and comparable timing characteristics, it seems plausible to transfer these calculations to the measurement of SCRs in fast event-related paradigms. We used optseq2 to generate individual stimulus sequences for each experimental session. This software first randomizes the sequence of events (without restrictions) and then introduces random jitter between events such that no ISI is shorter than the previously defined minimum ISI (2400 ms in this study) and the whole sequence duration does not exceed the previously defined maximum length (5 min in this study). This process is iteratively repeated and the design efficiency is stored for each sequence. We used 10,000 iterations and saved the best (in terms of design efficiency) 120 sequences (six sessions for each of 20 subjects) for later use. The optimization procedure as implemented in optseq2 does not make strong predictions about the shape of event-related responses since it does not use a template of the hemodynamic response function but instead a finite impulse response function spanning a predefined time window (0–8 s in this study). Thus, it is only assumed that the response morphology of stimulus-related responses is adequately described by the signal change within 8 s after stimulus presentation and stable across trials of one condition. Both these assumptions seem reasonable for stimulus-related SCRs that have a typical latency of 1–3 s, a rise time of 1–3 s (Dawson et al., 2007) and a response shape that is relatively stable within one examinee (Lim et al., 1997). With the currently chosen timing parameters, mean ISI amounted to 4952 ms (SD = 30 ms) across examinees. The average maximum ISI was 16230 ms (SD = 2323 ms) across examinees.

After completing the test, all participants were required to recall the probe items in a post-experimental memory test by means of a multiple-choice procedure. This test consisted of all items that were used in the CIT examination and participants were asked to identify the critical detail within each question. Finally, all participants were paid and fully debriefed about the nature of the study and the mock crime.

### Data acquisition and analysis

The electroencephalogram (EEG) was recorded continuously with a SynAmps amplifier (NeuroScan, Sterling, VA) from 19 cap-mounted Ag/AgCl electrodes (EasyCap, Germany) with positions according to the international 10–20 system; the reference electrode was placed at the right mastoid. To control for eye movements, vertical and horizontal electro-oculograms (EOG) were recorded. Data were digitized at 250 Hz and online filtered using a 0.05–40 Hz bandpass and a 50-Hz notch. Trials with eye blinks or eye movements (i.e., whenever the standard deviation within a 200-ms interval exceeded 30 μV in the horizontal or vertical EOG) as well as erroneous trials were excluded from further analyses. For central details, the number of valid trials amounted to 112.0 irrelevant (SD = 7.8), 28.2 probe (SD = 2.0) and 26.0 (SD = 3.1) target trials. The respective trial numbers for peripheral details were 112.8 irrelevant (SD = 8.7), 28.3 probe (SD = 1.8) and 25.4 (SD = 3.3) target trials. The amount of valid trials did not differ significantly between central and peripheral CIT questions (all *p* > 0.25 in paired *t*-tests contrasting the number of irrelevant, probe and target trials, respectively). The ERPs were separately computed for the three item types of central and peripheral CIT questions, with a time window ranging from −200 to 1400 ms relative to the visual stimulus onset. The 200-ms pre-stimulus interval served as baseline.

Similar to our previous work (Gamer and Berti, 2010), we determined the N200 amplitude by computing the maximally negative segment average of 50 ms at Cz within a time window ranging from 200 to 350 ms after stimulus onset. P300 amplitudes were calculated using the peak-to-peak method as described by Rosenfeld (Rosenfeld et al., 1991; Soskins et al., 2001) and used by a number of previous studies (e.g., Rosenfeld et al., 2004; Meijer et al., 2007; Verschuere et al., 2009). In a first step, the maximal positive 100 ms segment average was determined in a time-window ranging from 300 to 800 ms. Subsequently, the maximal negative 100 ms segment between the latency of the positive peak and 1400 ms was determined. Peak-to-peak P300 amplitude was defined as the difference between these two segments. This method was shown to be superior for the detection of concealed knowledge than the traditional base-to-peak measure (Soskins et al., 2001). As the P300 is most pronounced at Pz, amplitude calculations were limited to this site.

Skin conductance was measured by a constant voltage system (0.5 V) using a bipolar recording with two Ag/AgCl electrodes (0.8 cm diameter) filled with 0.05 M NaCl electrolyte. The electrodes were attached to the thenar and hypothenar eminences of the left hand. Skin conductance was digitized at 10 Hz and stored on an IBM PC for offline analysis.

To determine the amplitude of stimulus-related SCRs, we decomposed the skin conductance tracing into tonic and phasic components using an individually fitted template of a discrete SCR for each participant (Lim et al., 1997). In a first step, the algorithm that was implemented using the statistical programming language R (http://www.r-project.org) generated a template to match the individual SCR morphology. This template was optimized by minimizing the squared difference between the measured electrodermal data and the modeled response. In a second step, this SCR template was fitted to the whole skin conductance tracing of the respective participant. Additional SCRs were added when the model fit related to its complexity increased which was quantified using the Bayesian information criterion. The procedure resulted in a set of SCRs for each electrodermal recording that best resembled the measured data. Subsequently, SCRs that were elicited by the stimuli were identified by searching for responses with an onset between 1 and 3 s after stimulus onset. The amplitudes of these responses were finally log-transformed using the natural logarithm (Venables and Christie, 1980). To allow for a meaningful comparison of the ERPs with the electrodermal and behavioral data, we used the same trial selection as described above for these data channels.

To examine the effects of question (central vs. peripheral) and item type (probe vs. irrelevant) on the behavioral, electrodermal, and ERP data, we conducted a series of repeated measures analyses of variance (ANOVAs) on the corresponding dependent variables. Responses to target items were not included in the analyses since these items are typically not used to detect concealed knowledge (Meijer et al., 2007) and they were only included in the current study to ensure that participants are paying attention to the stimulus presentation. In addition to the factorial analyses, we also examined the interrelation between response systems. To this aim, we first computed differences between SCR, N200, and P300 amplitudes to probes and irrelevant items separately for central and peripheral details as well as for the whole test and subsequently calculated correlation coefficients between these measures.

In a second set of analyses, we tested whether the ISI affected SCR and ERP measures. To this aim, we separately averaged physiological responses for trials that were preceded by a short or a long ISI using a median split of the ISI distribution within each examinee. Ties were broken at random. The average ISIs amounted to 3236 ms (SD = 64 ms) for short ISIs and 6698 ms (SD = 104 ms) for long ones. After splitting the ISI distribution, we averaged the responses for irrelevant, probe and target details. Responses to central and peripheral questions were pooled in this analysis to have sufficient trials for ERP averaging. Finally, we conducted a series of repeated measures ANOVAs on these values using ISI (short vs. long) and item type (probe vs. irrelevant) as factors.

A rejection region of *p* < 0.05 was used for all statistical tests but effects yielding *p*-values below 0.10 are mentioned as marginally significant effects. Cohen's *f* (Cohen, 1988) is reported as an effect size estimate for ANOVA results.

## Results

### Post-experimental memory test

All participants remembered both central details (recognition rate *M* = 100%) and all but two participants additionally recognized both peripheral details (*M* = 95%, SD = 15%). The participants who forgot crime-related information only recognized one of two peripheral details. No statistically significant difference was observed between the memory for central and peripheral details, *t*_(19)_ = 1.45, *p* = 0.16. The whole pattern of results that is mentioned in the following does not change when confining the analyses to the 18 participants with perfect recognition of central and peripheral crime details.

### Behavioral responses

The ANOVA on the proportion of correct responses yielded a significant main effect of question type, [*F*_(1, 19)_ = 5.81, *p* = 0.03, *f* = 0.18], indicating that responses within the set of peripheral CIT questions were slightly more accurate (see Table 1). The main effect of item type, [*F*_(1, 19)_ = 2.78, *p* = 0.11, *f* = 0.23], as well as the interaction, [*F*_(1, 19)_ = 2.10, *p* = 0.16, *f* = 0.13], failed to reach statistical significance.

**Table 1:** **Average proportion of correct responses and reaction times for central and peripheral mock crime details as a function of item type**.

**Question type**	**Item type**	**Correct responses *M* (SD)**		**Reaction time (ms) *M* (SD)**
Central	Irrelevant	99.6% (0.6%)		534.7 (45.7)
	Probe	98.7% (2.0%)		592.3 (57.9)
	Target	90.0% (7.4%)		636.2 (61.4)
Peripheral	Irrelevant	99.8% (0.5%)		566.7 (63.2)
	Probe	99.5% (1.6%)		600.7 (78.6)
	Target	91.0% (6.4%)		663.8 (78.7)

Note: Mean and standard deviation for the proportion of correct responses were calculated for all trials of the corresponding experiment. In contrast, for reaction times, these values were computed on the basis of all valid responses that were selected for each question and item type (see text).

With respect to the response times, we observed a statistically significant main effect of item type, [*F*_(1, 19)_ = 29.53, *p* < 0.001, *f* = 0.37]. Responses to probes were slower than responses to irrelevant items and this effect seemed to be more pronounced for central details as indicated by a marginally significant interaction of question and item type, [*F*_(1, 19)_ = 4.33, *p* = 0.05, *f* = 0.09]. Moreover, response times tended to be longer for peripheral questions on average, [*F*_(1, 19)_ = 4.32, *p* = 0.05, *f* = 0.15] (see Table 1).

### Physiological measures: effects of question type

**Figure 2:**
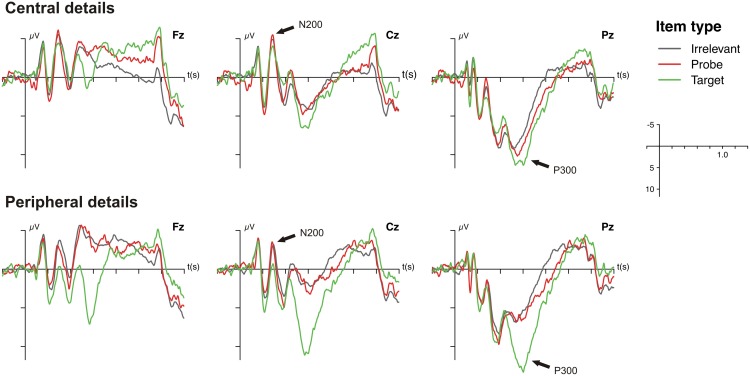
**Grand average event-related brain potentials (ERPs) as a function of question set (central vs. peripheral) and item type (target, probe, irrelevant) at Fz, Cz, and Pz.** Voltage and time scales are depicted on the right side.

**Figure 3:**
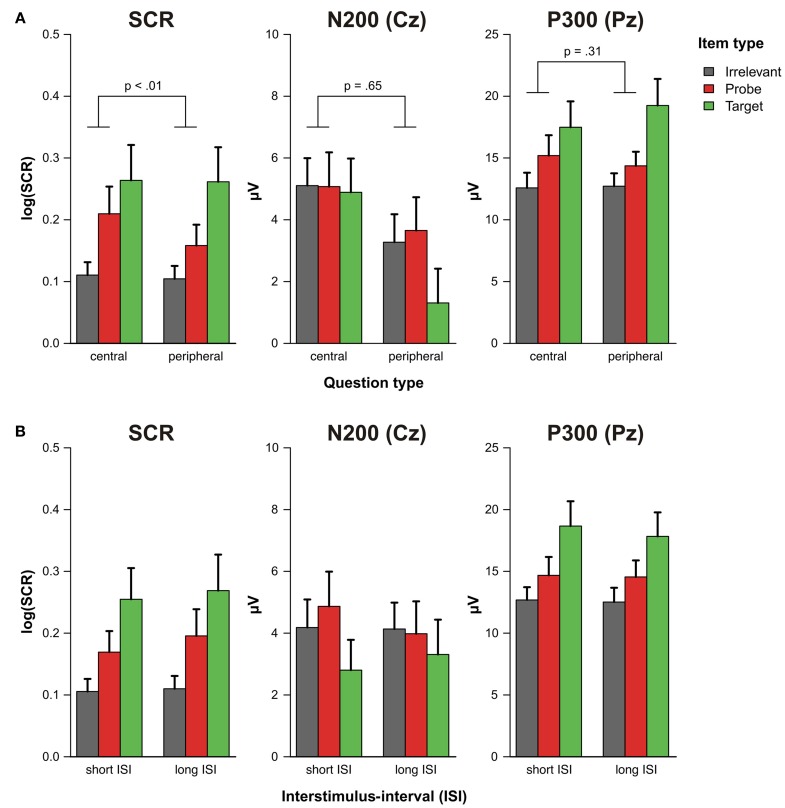
**Log-transformed skin conductance response amplitudes, N200 and P300 amplitudes (A) as a function of question set (central vs. peripheral) and item type (target, probe, irrelevant) and (B) as a function of interstimulus-interval (short vs. long) and item type (target, probe, irrelevant).** The *p*-value of the question × item type interaction excluding target items (see main text) is depicted above the corresponding bars in panel **(A)**. Error bars indicate standard errors of the mean.

As can be seen in the grand average ERPs that are depicted in Figure 2, all items were associated with a prominent P300 at Pz that was most pronounced for targets. The ANOVA on the P300 amplitudes revealed a significant main effect of item type, [*F*_(1, 19)_ = 28.90, *p* < 0.001, *f* = 0.19], indicating that P300 amplitudes to probes were larger than to irrelevant items (see Figure 3A). The main effect of question type, [*F*_(1, 19)_ = 0.18, *p* = 0.68, *f* = 0.03], and the interaction of question and item type were not statistically significant, [*F*_(1, 19)_ = 1.09, *p* = 0.31, *f* = 0.04].

N200 effects were also evident in the grand average ERPs (Figure 2) but seemed to be comparable between item types. The statistical analysis of the N200 amplitudes only revealed a significant main effect of question type, [*F*_(1, 19)_ = 8.22, *p* < 0.01, *f* = 0.19], indicating that central details were accompanied by an enhanced N200 (see Figure 3A). Neither the main effect of item type, [*F*_(1, 19)_ = 0.12, *p* = 0.73, *f* = 0.02], nor the interaction of both factors reached statistical significance, [*F*_(1, 19)_ = 0.21, *p* = 0.65, *f* = 0.02].

The response pattern of electrodermal responses was slightly different than that of the ERP measures. The ANOVA also yielded a significant main effect of item type, [*F*_(1, 19)_ = 13.18, *p* < 0.01, *f* = 0.28], indicating that SCRs were larger for probes as compared to irrelevant items. However, we additionally obtained a significant interaction of question and item type, [*F*_(1, 19)_ = 9.20, *p* < 0.01, *f* = 0.08], demonstrating that differential SCR amplitudes were more pronounced for central CIT questions (see Figure 3A). Overall, a significant main effect of question type, [*F*_(1, 19)_ = 5.74, *p* = 0.03, *f* = 0.10], further indicates that SCR amplitudes were larger within the set of central CIT questions.

Correlations between SCR, N200, and P300 amplitude differences contrasting probes and irrelevant CIT items were not significant when splitting the question set into central and peripheral items (Table 2). Only when pooling responses across the whole test, moderate correlations between SCR amplitudes on the one hand and N200 as well as P300 amplitudes on the other hand emerged. Importantly, positive associations were observed which resemble the expected pattern for SCR and P300 amplitudes. However, since larger N200 amplitudes were thought to index probe recognition (Matsuda et al., 2009; Gamer and Berti, 2010), a positive correlation between SCR and N200 amplitudes was not expected.

**Table 2:** **Intercorrelations between the response differences of all physiological measures for central and peripheral mock crime details as well as for the whole test**.

	**N200**	**P300**
**CENTRAL QUESTIONS**		
SCR	0.35 (0.13)	0.33 (0.15)
N200	−	0.02 (0.92)
**PERIPHERAL QUESTIONS**		
SCR	0.13 (0.59)	0.15 (0.54)
N200	−	0.05 (0.82)
**WHOLE TEST**		
SCR	0.45 (0.04)	0.45 (0.04)
N200	−	0.16 (0.50)

Note: Values in brackets indicate p-values derived from tests for significant differences from r = 0 (N = 20). SCR, skin conductance response.

### Physiological measures: effects of ISI

The pattern of the SCR and the ERP responses was highly similar irrespective of whether a trial was preceded by a relatively short or long ISI (Figure 3B). The ANOVA on the P300 amplitudes yielded a significant main effect of item type, [*F*_(1, 19)_ = 21.42, *p* < 0.001, *f* = 0.18], indicating larger responses to probes as compared to irrelevant items. Neither the main effect of ISI, [*F*_(1, 19)_ = 0.15, *p* = 0.71, *f* = 0.01], nor the interaction of both factors reached statistical significance, [*F*_(1, 19)_ < 0.01, *p* = 0.96, *f* < 0.01].

In the ANOVA on the N200 amplitudes, we did not obtain any significant effect: Main effect of item type, [*F*_(1, 19)_ = 0.33, *p* = 0.57, *f* = 0.03], main effect of ISI, [*F*_(1, 19)_ = 0.64, *p* = 0.43, *f* = 0.05], interaction of both factors, [*F*_(1, 19)_ = 0.76, *p* = 0.39, *f* = 0.05].

For the electrodermal responses, the ANOVA yielded a significant main effect of item type, [*F*_(1, 19)_ = 13.66, *p* < 0.01, *f* = 0.28], demonstrating larger SCR amplitudes to probes than to irrelevant items. The main effect of ISI, [*F*_(1, 19)_ = 2.31, *p* = 0.14, *f* = 0.05], as well as the interaction of ISI and item type, [*F*_(1, 19)_ = 0.97, *p* = 0.34, *f* = 0.04], failed to reach statistical significance.

## Discussion

The current study aimed at examining whether depth of processing differentially affects electrodermal and ERP measures in a CIT. Although crime related details were only incidentally encoded, participants showed very high recognition rates of central and peripheral crime details in a memory test that was conducted one week after the mock crime. Since recognition memory did not differ significantly between question types, any differences in behavioral or physiological responses between central and peripheral crime details could not be attributed to differences in explicit memory.

Consistent with previous studies using a comparable CIT protocol, we observed longer response times for probes as compared to irrelevant items (Farwell and Donchin, 1991; Seymour et al., 2000; Gamer and Berti, 2010). This effect tended to be more pronounced for central details. Moreover, we obtained an overall trend for longer response times in the set of peripheral details, which might indicate that the response selection task was more difficult for this type of information. However, this effect could also result from a speed-accuracy tradeoff since response accuracy was also higher for peripheral crime details.

Electrodermal responses were significantly larger for probes than for irrelevant items but this difference was more pronounced for central mock crime details. This pattern of results replicates previous studies showing enhanced electrodermal responses to central as compared to peripheral crime details (Nahari and Ben-Shakhar, 2011; Peth et al., 2012) even in the absence of differences in explicit memory (Gamer et al., 2012). Thus, it seems that SCRs are sensitive to depth of processing.

In this study, we expanded current research by also testing whether an optimization of the experimental stimulation in terms of item sequence and ISIs (Dale, 1999) would improve the quantification of SCRs while keeping the whole duration of the experiment sufficiently short. It was recently shown that ISIs can be reduced to 10 s without reducing CIT validity (Breska et al., 2010). In the current study, the mean ISI was as short as 5 s but we still observed substantial differences in SCR amplitudes between probes and irrelevant items. Moreover, the ISI did not have a significant influence on this response pattern. Thus, SCR amplitudes were stable irrespective of whether a given stimulus was preceded by a relatively short (~3.2 s) or long (~6.7 s) ISI. Taken together, our procedure seems to be a viable method for future studies requiring short ISIs because of a simultaneous measurement of electrodermal responses and ERPs.

Consistent with previous studies, we observed larger P300 amplitudes for probes as compared to irrelevant items (Rosenfeld, 2011). Interestingly however, this effect was similar for central and peripheral crime details. Thus, in contrast to electrodermal responses, this measure might be less affected by depth of processing and seems to primarily reflect successful item recognition (Meijer et al., 2009). This result is at odds with previous studies showing enhanced P300 responses to deeply encoded episodic or autobiographical information as compared to incidentally acquired knowledge or shallowly encoded details (Ferlazzo et al., 1993; Ellwanger et al., 1996; Rosenfeld et al., 2006, 2007). However, the autobiographical information that was used in these previous studies was very salient and highly relevant to the participant. Such information might not be representative for episodic memories even when they concern deeply encoded details of high personal relevance (e.g., a weapon that was used in a murder). Importantly, the current data does not suggest that a large number of peripheral details could be included in a CIT examination without affecting the validity of P300 amplitudes for detecting concealed knowledge. Since peripheral details are usually remembered less well especially when the CIT is conducted weeks or even months after the crime, it is likely that CIT validity will drop when including such details in the examination (Gamer et al., 2010; Nahari and Ben-Shakhar, 2011; Peth et al., 2012). In the current study, there was no difference in explicit memory and only under these circumstances, P300 amplitudes seem to reflect successful recognition instead of encoding depth.

Unexpectedly, ISI did not influence P300 amplitudes in the current study, and response differences between probes and irrelevant items were stable irrespective of the preceding ISI. Previous studies showed that P300 amplitudes depend on stimulus probability, stimulus sequence structure and ISI (Polich and Bondurant, 1997; Sambeth et al., 2004). All these factors affect the target-to-target interval (TTI) and it has been demonstrated that the TTI is indeed the major determinant of P300 amplitudes (Gonsalvez et al., 1999; Gonsalvez and Polich, 2002). Moreover, it has been described that the temporal structure of events affects P300 amplitudes even when TTI is kept constant (Schwartze et al., 2011). Thus, a random ISI as used in the current study should reduce P300 amplitudes. However, we did neither observe an effect of ISI on P300 amplitudes, not did we obtain reduced P300 responses. By contrast, the overall pattern of P300 amplitudes in the current study as well as their size were very similar to previous CIT studies using a short, fixed ISI (e.g., Rosenfeld et al., 2006, 2007; Verschuere et al., 2009). It seems possible that effects of the preceding ISI were reduced in the current study because of the use of random ISIs. Thus, even though the timing between successive stimuli was highly variable in the current study, the interval between stimuli of the same category (i.e., probe, target, irrelevant item) was relatively stable due to the random nature of the stimulus sequence. Therefore, the overall influence of our session structure and timing on P300 amplitudes might have been less pronounced as compared to previous studies using different sets of fixed ISIs to examine predictors of P300 amplitudes (e.g., Polich, 1990). Moreover, the mean ISI in the current study (4952 ms) was much longer as compared to a previous study reporting a reduction of P300 amplitudes for random as compared to isochronous sequences (900 ms, Schwartze et al., 2011). Thus, it seems possible that effects of the temporal structure of stimulation are less pronounced when using larger ISIs. Nevertheless, it would be interesting and important for future studies to examine the influence of ISI structure (random vs. fixed) and length on P300 amplitudes in the CIT in more detail.

In contrast to other studies, we did not observe differences in N200 amplitudes between crime details and irrelevant CIT items (Matsuda et al., 2009; Gamer and Berti, 2010). The N200 has previously been linked to response monitoring demands as well as to the orienting of attentional resources (for a review see Folstein and Van Petten, 2008). Thus, it was reasoned that enhanced N200 amplitudes to crime related information in the CIT might index the automatic orienting of attention toward probe items in order to facilitate a more extensive processing of such personally relevant information (Matsuda et al., 2009). Additionally, it was suggested that the N200 reflects enhanced response monitoring as a pre-requisite for correctly responding to probes that pop out of the stimulus stream (as targets do) but usually require a different behavioral response (Gamer and Berti, 2010). Both these circumstances also apply to the current study but we did not obtain differences in N200 amplitudes between crime related and irrelevant details. However, the present study differs from previous experiments with respect to the stimulus timing (i.e., the ISI) as well as to the stimuli that were used in the CIT: In line with recommendations for the field use of the CIT, we constructed our item set in such a way that all items were clearly separable from each other but equally plausible for an innocent examinee (Nakayama, 2002; Meijer et al., 2011). By contrast, in the study by Gamer and Berti (2010), the visual stimuli presented as probes and targets were perceptually less distinct (for instance, the jack of spades vs. the king of spades from a set of playing cards). Matsuda et al. (2009) used spoken digits that also form a more homogeneous stimulus set than the natural visual objects used in the present study. Indeed, it has been reported that N200 effects are modulated by the perceptual overlap of stimuli that require different behavioral responses (Nieuwenhuis et al., 2004): When stimuli were clearly separable, the N200 was substantially reduced. Therefore, a lack of an effect of item type in our results might be attributed to the fact that crime details and irrelevant CIT items were easily separable in the current study and presumably did not require additional processing demands. This is in line with the functional interpretation by Folstein and Van Petten (2008), supposing that the fronto-central N200 is a correlate of cognitive control, reflecting for instance attentional allocation to one (Gramann et al., 2007) or different behaviorally relevant visual dimensions (Berti and Wühr, 2012). Since we did not observe a differential N200 between crime related and irrelevant items but instead a general difference between central and peripheral CIT questions, it seems that the N200 does not mirror processing of crime related information per se but is more dependent on stimulus characteristics. In line with previous studies, ISI did not affect N200 amplitudes (Polich, 1990), but it seems that certain features of the stimulus set modulated N200 amplitudes. Since we have no further interpretation of why N200 responses were generally enhanced for central details, we propose that further investigations have to determine when enhanced N200 amplitudes to specific items can be expected in the CIT.

To sum up, the present study revealed a differential sensitivity of ERP measures and electrodermal responses to depth of processing. We observed larger SCRs for central items along with stable P300 responses across question types. This differential sensitivity of response systems might be one reason for the small correlations between measures (Matsuda et al., 2009; Gamer and Berti, 2010) and the incremental validity that has been reported previously (Ambach et al., 2010; Matsuda et al., 2011). Thus, also from an applied perspective, it seems useful to combine different physiological measures that cover partly different psychological processes that are all involved in CIT examinations (e.g., item recognition, attentional orienting, response selection and monitoring). An important question for future research is the identification and characterization of these processes that might differentially affect autonomic (Ambach et al., 2008; Gamer et al., 2008) and central nervous system responses in the CIT (Gamer and Berti, 2010).

### Conflict of interest statement

The authors declare that the research was conducted in the absence of any commercial or financial relationships that could be construed as a potential conflict of interest.

## References

[B1] Abootalebi V. Moradi M. H. Khalilzadeh M. A. (2006). A comparison of methods for ERP assessment in a P300-based GKT. Int. J. Psychophysiol. 62, 309–320 10.1016/j.ijpsycho.2006.05.009 16860894

[B2] Allen J. J. Iacono W. G. Danielson K. D. (1992). The identification of concealed memories using the event-related potential and implicit behavioral measures: a methodology for prediction in the face of individual differences. Psychophysiology 29, 504–522 1410180 10.1111/j.1469-8986.1992.tb02024.x

[B3] Ambach W. Bursch S. Stark R. (2010). A Concealed Information Test with multimodal measurement. Int. J. Psychophysiol. 75, 258–267 10.1016/j.ijpsycho.2009.12.007 20026133

[B4] Ambach W. Dummel S. Lüer T. Vaitl D. (2011). Physiological responses in a Concealed Information Test are determined interactively by encoding procedure and questioning format. Int. J. Psychophysiol. 81, 275–282 10.1016/j.ijpsycho.2011.07.010 21803080

[B5] Ambach W. Stark R. Peper M. Vaitl D. (2008). Separating deceptive and orienting components in a Concealed Information Test. Int. J. Psychophysiol. 70, 95–104 10.1016/j.ijpsycho.2008.07.002 18674573

[B6] Ben-Shakhar G. Bar-Hillel M. Kremnitzer M. (2002). Trial by polygraph: reconsidering the use of the guilty knowledge technique in court. Law Hum. Behav. 26, 527–541 12412496 10.1023/a:1020204005730

[B7] Ben-Shakhar G. Elaad E. (2003). The validity of psychophysiological detection of information with the Guilty Knowledge Test: a meta-analytic review. J. Appl. Psychol. 88, 131–151 12675401 10.1037/0021-9010.88.1.131

[B8] Berti S. Wühr P. (2012). Using redundant visual information from different dimensions for attentional selection. J. Psychophysiol. 26, 99–104

[B9] Breska A. Maoz K. Ben-Shakhar G. (2010). Interstimulus intervals for skin conductance response measurement. Psychophysiology 48, 437–440 10.1111/j.1469-8986.2010.01084.x 20701710

[B10] Carmel D. Dayan E. Naveh A. Raveh O. Ben-Shakhar G. (2003). Estimating the validity of the Guilty Knowledge Test from simulated experiments: the external validity of mock crime studies. J. Exp. Psychol. Appl. 9, 261–269 10.1037/1076-898X.9.4.261 14664677

[B11] Cohen J. (1988). Statistical Power Analysis for the Behavioral Sciences, 2nd Edn Hillsdale, NJ: Erlbaum

[B12] Craik F. I. M. Tulving E. (1975). Depth of processing and the retention of words in episodic memory. J. Exp. Psychol. 104, 268–294

[B13] Dale A. M. (1999). Optimal experimental design for event-related fMRI. Hum. Brain Mapp. 8, 109–114 10.1002/(SICI)1097-0193(1999)8:2/3<109::AID-HBM7>3.0.CO;2-W 10524601 PMC6873302

[B14] Dawson M. E. Schell A. M. Filion D. L. (2007). The electrodermal system, in Handbook of Psychophysiology, eds Cacioppo J. T. Tassinary L. G. Berntson G. G. (Cambridge, MA: University Press), 159–181

[B15] Ellwanger J. Rosenfeld J. P. Sweet J. J. Bhatt M. (1996). Detecting simulated amnesia for autobiographical and recently learned information using the P300 event-related potential. Int. J. Psychophysiol. 23, 9–23 8880362 10.1016/0167-8760(96)00035-9

[B16] Farwell L. A. Donchin E. (1991). The truth will out: interrogative polygraphy (“lie detection”) with event-related brain potentials. Psychophysiology 28, 531–547 1758929 10.1111/j.1469-8986.1991.tb01990.x

[B17] Ferlazzo F. Conte S. Gentilomo A. (1993). Event-related potentials and recognition memory within the “levels of processing” framework. Neuroreport 4, 667–670 8347805 10.1097/00001756-199306000-00016

[B18] Folstein J. R. Van Petten C. (2008). Influence of cognitive control and mismatch on the N2 component of the ERP: a review. Psychophysiology 45, 152–170 10.1111/j.1469-8986.2007.00602.x 17850238 PMC2365910

[B19] Gamer M. (2011). Detecting concealed information using autonomic measures, in Memory Detection: Theory and Application of the Concealed Information Test, eds Verschuere B. Ben-Shakhar G. Meijer E. H. (Cambridge, MA: University Press), 27–45

[B20] Gamer M. Berti S. (2010). Task relevance and recognition of concealed information have different influences on electrodermal activity and event-related brain potentials. Psychophysiology 47, 355–364 10.1111/j.1469-8986.2009.00933.x 20003148

[B21] Gamer M. Gödert H. W. Keth A. Rill H.-G. Vossel G. (2008). Electrodermal and phasic heart rate responses in the Guilty Actions Test: comparing guilty examinees to informed and uninformed innocents. Int. J. Psychophysiol. 69, 61–68 10.1016/j.ijpsycho.2008.03.001 18433904

[B22] Gamer M. Klimecki O. Bauermann T. Stoeter P. Vossel G. (2012). fMRI-activation patterns in the detection of concealed information rely on memory-related effects. Soc. Cogn. Affect. Neurosci. 7, 506–515 10.1093/scan/nsp005 19258375 PMC3375883

[B23] Gamer M. Kosiol D. Vossel G. (2010). Strength of memory encoding affects physiological responses in the Guilty Actions Test. Biol. Psychol. 83, 101–107 10.1016/j.biopsycho.2009.11.005 19931347

[B24] Gonsalvez C. J. Gordon E. Grayson S. Barry R. J. Lazzaro I. Bahramali H. (1999). Is the target-to-target interval a critical determinant of P3 amplitude? Psychophysiology 36, 643–654 10442033

[B25] Gonsalvez C. L. Polich J. (2002). P300 amplitude is determined by target-to-target interval. Psychophysiology 39, 388–396 10.1017/S0048577201393137 12212658

[B26] Gramann K. Toellner T. Krummenacher J. Eimer M. Müller H. J. (2007). Brain electrical correlates of dimensional weighting: an ERP study. Psychophysiology 44, 277–292 10.1111/j.1469-8986.2007.00496.x 17343711

[B27] Lim C. L. Rennie C. Barry R. J. Bahramali H. Lazzaro I. Manor B. (1997). Decomposing skin conductance into tonic and phasic components. Int. J. Psychophysiol. 25, 97–109 10.1016/S0167-8760(96)00713-1 9101335

[B28] Lykken D. T. (1959). The GSR in the detection of guilt. J. Appl. Psychol. 43, 385–388

[B29] Matsuda I. Hirota A. Ogawa T. Takasawa N. Shigemasu K. (2006). A new discrimination method for the Concealed Information Test using pretest data and within-individual comparisons. Biol. Psychol. 73, 157–164 10.1016/j.biopsycho.2006.01.013 16504367

[B30] Matsuda I. Nittono H. Hirota A. Ogawa T. Takasawa N. (2009). Event-related brain potentials during the standard autonomic-based concealed information test. Int. J. Psychophysiol. 74, 58–68 10.1016/j.ijpsycho.2009.07.004 19631702

[B31] Matsuda I. Nittono H. Ogawa T. (2011). Event-related potentials increase the discrimination performance of the autonomic-based concealed information test. Psychophysiology 48, 1701–1710 10.1111/j.1469-8986.2011.01266.x 21806637

[B32] Meijer E. H. Smulders F. T. Merckelbach H. L. Wolf A. G. (2007). The P300 is sensitive to concealed face recognition. Int. J. Psychophysiol. 66, 231–237 10.1016/j.ijpsycho.2007.08.001 17825933

[B33] Meijer E. H. Smulders F. T. Wolf A. (2009). The contribution of mere recognition to the p300 effect in a concealed information test. Appl. Psychophysiol. Biofeedback 34, 221–226 10.1007/s10484-009-9099-9 19585234 PMC2727362

[B34] Meijer E. H. Verschuere B. Ben-Shakhar G. (2011). Practical guidelines for developing a CIT, in Memory Detection: Theory and Application of the Concealed Information Test, eds Verschuere B. Ben-Shakhar G. Meijer E. H. (Cambridge, MA: University Press), 293–302

[B35] Mertens R. Allen J. J. (2008). The role of psychophysiology in forensic assessments: deception detection, ERPs, and virtual reality mock crime scenarios. Psychophysiology 45, 286–298 10.1111/j.1469-8986.2007.00615.x 17995914

[B36] Nahari G. Ben-Shakhar G. (2011). Psychophysiological and behavioral measures for detecting concealed information: the role of memory for crime details. Psychophysiology 48, 733–744 10.1111/j.1469-8986.2010.01148.x 20958308

[B37] Nakayama M. (2002). Practical use of the concealed information test for criminal investigation in Japan, in Handbook of Polygraph Testing, ed Kleiner M. (San Diego, CA: Academic Press), 49–86

[B38] Nieuwenhuis S. Yeung N. Cohen J. D. (2004). Stimulus modality, perceptual overlap, and the go/no-go N2. Psychophysiology 41, 157–160 10.1046/j.1469-8986.2003.00128.x 14693011

[B39] Peth J. Vossel G. Gamer M. (2012). Emotional arousal modulates the encoding of crime-related details and corresponding physiological responses in the Concealed Information Test. Psychophysiology 49, 381–390 10.1111/j.1469-8986.2011.01313.x 22188567

[B40] Polich J. (1990). Probability and inter-stimulus interval effects on the P300 from auditory stimuli. Int. J. Psychophysiol. 10, 163–170 10.1016/0167-8760(90)90030-H 2272863

[B41] Polich J. Bondurant T. (1997). P300 sequence effects, probability, and interstimulus interval. Physiol. Behav. 61, 843–849 10.1016/S0031-9384(96)00564-1 9177555

[B42] Rosenfeld J. Angell A. Johnson M. (1991). An ERP-based, control-question lie detector analog: algorithms for discriminating effects within individuals' average waveforms. Psychophysiology 28, 319–335 1946897 10.1111/j.1469-8986.1991.tb02202.x

[B43] Rosenfeld J. P. (2011). P300 in detecting deception, in Memory Detection: Theory and Application of the Concealed Information Test, eds Verschuere B. Ben-Shakhar G. Meijer E. H. (Cambridge, MA: University Press), 63–89

[B44] Rosenfeld J. P. Biroschak J. R. Furedy J. J. (2006). P300-based detection of concealed autobiographical versus incidentally acquired information in target and non-target paradigms. Int. J. Psychophysiol. 60, 251–259 10.1016/j.ijpsycho.2005.06.002 16137781

[B45] Rosenfeld J. P. Ellwanger J. Sweet J. (1995). Detecting simulated amnesia with event-related brain potentials. Int. J. Psychophysiol. 19, 1–11 10.1016/0167-8760(94)00057-L 7790284

[B46] Rosenfeld J. P. Shue E. Singer E. (2007). Single versus multiple probe blocks of P300-based concealed information tests for self-referring versus incidentally obtained information. Biol. Psychol. 74, 396–404 10.1016/j.biopsycho.2006.10.002 17126984

[B47] Rosenfeld J. P. Soskins M. Bosh G. Ryan A. (2004). Simple, effective countermeasures to P300-based tests of detection of concealed information. Psychophysiology 41, 205–219 10.1111/j.1469-8986.2004.00158.x 15032986

[B48] Sambeth A. Maes J. H. Brankack J. (2004). With long intervals, inter-stimulus interval is the critical determinant of the human P300 amplitude. Neurosci. Lett. 359, 143–146 10.1016/j.neulet.2004.01.064 15050684

[B49] Schwartze M. Rothermich K. Schmidt-Kassow M. Kotz S. A. (2011). Temporal regularity effects on pre-attentive and attentive processing of deviance. Biol. Psychol. 87, 146–151 10.1016/j.biopsycho.2011.02.021 21382437

[B50] Seymour T. L. Seifert C. M. Shafto M. G. Mosmann A. L. (2000). Using response time measures to assess “guilty knowledge.” J. Appl. Psychol. 85, 30–37 10740954 10.1037/0021-9010.85.1.30

[B51] Soskins M. Rosenfeld J. P. Niendam T. (2001). Peak-to-peak measurement of P300 recorded at 0.3 Hz high pass filter settings in intraindividual diagnosis: complex vs. simple paradigms. Int. J. Psychophysiol. 40, 173–180 10.1016/S0167-8760(00)00154-9 11165356

[B52] van Hooff J. C. Brunia C. H. Allen J. J. (1996). Event-related potentials as indirect measures of recognition memory. Int. J. Psychophysiol. 21, 15–31 10.1016/0167-8760(95)00043-7 8839121

[B53] Venables P. H. Christie M. J. (1980). Electrodermal activity, in Techniques in Psychophysiology, eds Martin I. Venables P. H. (Chichester: Wiley), 3–67

[B54] Verschuere B. Ben-Shakhar G. Meijer E. H. (2011). Memory Detection: Theory and Application of the Concealed Information Test. Cambridge, MA: Cambridge University Press

[B55] Verschuere B. Rosenfeld J. P. Winograd M. R. Labkovsky E. Wiersema R. (2009). The role of deception in P300 memory detection. Leg. Crim. Psychol. 14, 253–262

[B56] Vrij A. (2008). Detecting Lies and Deceit. Pitfalls and Opportunities. Chichester: Wiley

